# Scrotal Apocrine Adenocarcinoma with Pagetoid Phenomenon and Inguinal Lymph Node Metastases

**DOI:** 10.1155/2016/8353745

**Published:** 2016-10-12

**Authors:** Aristeidis Hristos Zibis, Apostolos Haralampos Fyllos, Sophia Havaki, Sotirios Sotiriou, Georgios Kotakidis, Dimitrios Leonidas Arvanitis

**Affiliations:** ^1^Department of Anatomy, Faculty of Medicine, School of Health Sciences, University of Thessaly, Larissa, Greece; ^2^Department of Histology and Embryology, National and Kapodistrian University of Athens, Medical School, Athens, Greece; ^3^Department of Embryology, Faculty of Medicine, School of Health Sciences, University of Thessaly, Larissa, Greece; ^4^Department of Urology, General Hospital of Florina, Florina, Greece

## Abstract

We report a case of scrotal apocrine adenocarcinoma in a 72-year-old Caucasian male which was initially presented as a reddish superficial lesion which in time became an ulcerated nodule. The initial pathological examination showed an apocrine adenocarcinoma with pagetoid phenomenon. The tumor recurred after four months and then excision biopsy showed tumor with pagetoid phenomenon which reached all the surgical margins. Three months later an ulcerated nodule in the scrotum and greatly enlarged ipsilateral inguinal lymph nodes were noticed. The final pathological examination showed multiple separated malignant foci, some with overlying pagetoid phenomenon and inguinal lymph node metastases. Immunohistochemistry showed positivity for Gross Cystic Disease Fluid Protein-15 (GCDFP-15), androgen receptors, and score 3+ for the Human Epidermal growth factor Receptor-2 (HER2). The aggressive behavior of the present tumor goes along with previous reports showing that HER2 high score cases exhibit a worse prognosis.

## 1. Introduction

Scrotal apocrine adenocarcinoma is a rare and insidious malignancy with prolonged clinical course, because of belated diagnosis and its high probability for local recurrence. The incidence of extramammary cutaneous apocrine carcinoma (located mostly in axilla and the inguinal area due to their increased number of apocrine glands) ranges from 0.0049 to 0.0173/100 000 per year [1]. An apocrine gland carcinoma sometimes can show epidermotropism forming intraepidermal spread which is called pagetoid phenomenon, an uncommon feature. The difference between pagetoid phenomenon and Paget's disease is that in the former there is an underlying tumor which spreads upwards and infiltrates the epidermis with malignant cells which lie either isolated or in small clusters within the epidermis, whereas in Paget's disease there is no underlying tumor to begin with. In our case, the pagetoid phenomenon is derived from the underlying apocrine carcinoma, which is a rarely published entity [2, 3].

## 2. Case Presentation

The patient is a 72-year-old Caucasian male who first visited his dermatologist two years ago because he had noticed a small superficial red lesion on the right side of his scrotum. His dermatologist treated him with local ointments with no improvement. The lesion grew larger and became indurated and ulcerated. He was consequently referred to a urologist a year and a half after initial presentation, who decided to surgically remove the lesion and send it for histopathological examination.

**Figure 1:**
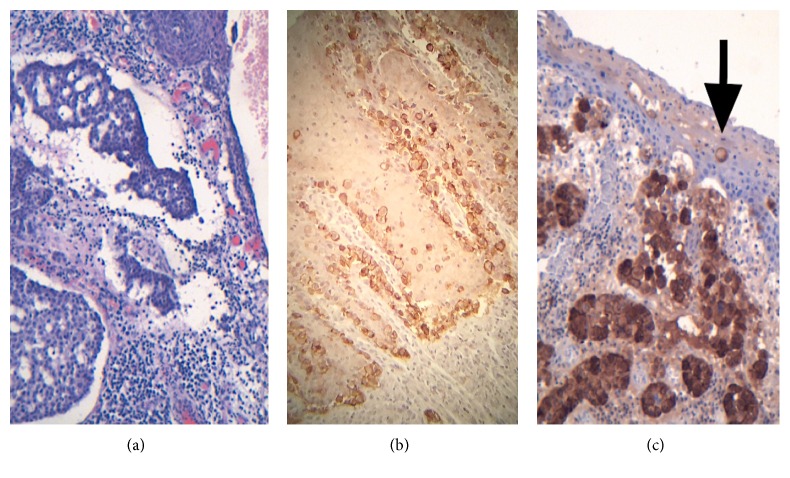
(a) Cribriform pattern of apocrine adenocarcinoma. Original magnification: ×100, hematoxylin/eosin stain. (b) Pagetoid infiltration of the epidermis by the malignant cells which show score 3+ staining for HER 2 in the lateral margin of the resection. Magnification: ×250. (c) Immunostaining for Gross Cystic Disease Fluid Protein-15 (GCDFP-15). Intense and moderate cytoplasmic staining of tumor cells. Pagetoid infiltration of the epidermis by one tumor cell (arrow). Original magnification: ×100.

**Figure 2:**
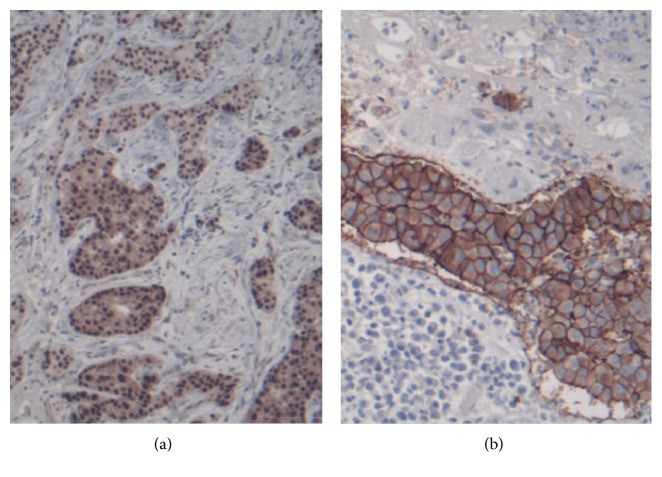
(a) Immunostaining for androgen receptor. Intense nuclear staining in the overwhelming majority of tumor cells. (b) Immunostaining for Human Epidermal growth factor Receptor-2 (HER2). Score 3+. Original magnification: ×100.

On gross pathological examination, the specimen consisted of a segment of the scrotum measuring 2 cm × 0.8 cm in surface area and 1.2 cm in depth, with an ulcerated surface lesion measuring 1.8 cm in maximum diameter. On dissection, a compact white indurated mass was underneath the ulcerated surface measuring 1.8 cm. Microscopic examination revealed an adenocarcinoma consisting of relatively large cells with abundant amphophilic to eosinophilic and locally clear cytoplasm, with large mainly open nuclei with large nucleoli, and with many mitoses. The malignant cells were arranged mainly in a cribriform pattern (Figure 1(a)) and less frequently in small compact tumor islands or cords or tubules. The adenocarcinoma cells infiltrated and ulcerated the overlying epidermis. At the borders of the ulcer, isolated or small clusters of tumor cells infiltrated the epidermis in a pagetoid pattern. The tumor cells infiltrated deep between the smooth muscle cells of dartos. A small number of tumor emboli in the lymphatic vessels were noticed. The infiltrating portion of the tumor was totally excised but the pagetoid infiltration reached both lateral margins (Figure 1(b)). Immunohistochemical staining revealed that almost all tumor cells had positive medium or intense cytoplasmic staining for Gross Cystic Disease Fluid Protein-15 (GCDFP-15) (Figure 1(c)), confirming the apocrine nature of the adenocarcinoma. In addition, almost all tumor cells showed intense nuclear staining for the androgen receptor (Figure 2(a)) and intense homogeneous cell membrane staining for the Human Epidermal growth factor Receptor-2 (HER2) (score 3+) (Figure 2(b)). Carcinoembryonic antigen (CEA) was negative. No internal malignancy was discovered after following metastatic protocol.

**Figure 3:**
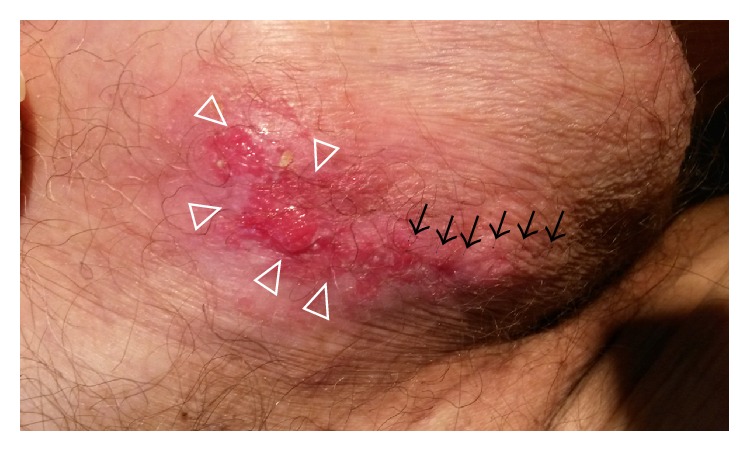
Macroscopic image of the scrotum. The arrows show the incision of the previous surgical treatment. The arrowheads show the area of local recurrence.

In a follow-up visit at 4 months postoperatively, there was a possible recurrence of the carcinoma. Macroscopically, it appeared as a red skin area around the ulceration (Figure 3). Microscopically, the recurrent tumor was composed of the same apocrine carcinoma cells as the initial tumor and in this recurrent site the tumor cells had formed many trabecular structures. Other observed features were few lymphatic tumor emboli, ulceration of the overlying epidermis, and pagetoid infiltration of the remaining epidermis around the ulcerated lesion. The tumor cells had reached all surgical margins. The patient refused regional lymph node exploration and any form of chemotherapy.

**Figure 4:**
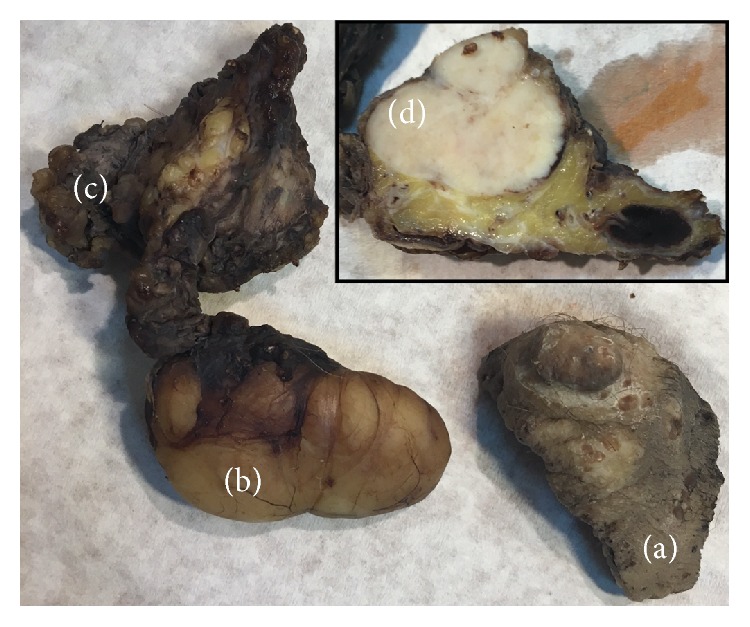
A composite picture showing (a) a recurrent nodule on the surface of the excised scrotal segment (low right part); (b) the incidental lipoma (low left part); (c) the fibrofatty inguinal tissues (upper left part); (d) cut surface of the fibrofatty tissues showing the largest inguinal lymph node being replaced totally by the white malignant metastatic tumor (inset right upper part).

3.5 months later, the patient developed an ulcerated nodule 1.5 cm in diameter on the scrotum near the previous surgical scar which was excised (Figure 4). Excision of the enlarged ipsilateral inguinal lymph nodes also took place. The histopathological examination revealed foci of malignant cells spread in the segment of the scrotum. The macroscopic nodule was ulcerated. There were several separate foci of malignant cells, some in the deep parts of the scrotum and others in the superficial parts of the scrotum. These superficial foci showed upward infiltration of the epidermis as a pagetoid phenomenon. In this specimen there were many lymphatic tumor emboli. In the fibrofatty tissues of the inguinal region 16 lymph nodes were found, with the largest one measuring 3.5 cm in diameter with a white firm cut surface (Figure 4). An adjacent incidental lipoma measuring 6 cm in diameter was excised. Microscopic examination revealed malignant metastasis in 11 lymph nodes with the biggest one being totally replaced by malignant cells which extended beyond the lymph node capsule in the surrounding fibrofatty tissues.

## 3. Discussion

Symptomatology of apocrine scrotal carcinoma is extremely nonspecific leading to delay in diagnosis. It is a slow growing cancer often manifested as scrotal eczema and/or dermatitis with pruritus. Solid or cystic masses, reddish or purple subcutaneous nodules of different sizes ranging up to occupying the whole of the scrotum, are also features of the disease. Adding up to the confusion and variation of the clinical presentation is the prolonged use of local corticosteroids or antifungal agents before correct diagnosis. Therefore, it may be confused with superficial spreading melanoma, Bowen's disease, the epidermal phase of neuroendocrine carcinoma, mycosis fungoides, psoriasis, leukoplakia, superficial fungal infection, and clear-cell papulosis. Confirmation of diagnosis can only be made by excision of the lesion and histological examination. It has metastatic potential to regional lymph nodes, lungs, liver, brain, and bone, while multiple local recurrences are not unheard of [1–4]. Patients with lymph node metastasis have a diminished survival, which justifies frequent follow-up visits [1]. Positive surgical margins are one of the most important risk factors for local recurrence. Interestingly, in our case, whereas the infiltrating component was initially completely removed with free surgical margins, the recurrence had again an infiltrated component. This can be explained by either translymphatic implantations in the adjacent areas of the scrotum or multifocal development of the tumor (the other infiltrated areas were not developed enough at the time of the first excision). It must be taken into consideration that the horizontal pagetoid infiltration spreads more laterally beyond the underlying infiltrated component and therefore wider surgical excision must be contemplated.

A recent study proposes preoperative histological examination by multiple sharp punch biopsies from 2 cm or more around the peripheries of the lesions at short intervals, in order to increase the reliability of pathological examination and identify the margins of the lesion. The mean delay time from the onset of symptoms to diagnosis did not show any statistical relationship with dermal invasion of Paget's cells, which plays a critical part for distant metastasis ability of the tumor [5]. Regional lymph node dissection must be considered in the presence of clinically positive nodes [1], as in our case.

HER2-positive apocrine carcinoma cells have increased invasive and metastatic potential. Recently, HER2 positivity was significantly correlated with multiple lymph node metastases but was not correlated with the patients' sex, tumor size, nodule formation, or margin status. The incidence of cases with HER2 scores of 2+ or 3+ was higher in cases of invasive extramammary Paget's disease (EMPD) than in intraepithelial EMPD. The proposed molecular mechanism responsible is that HER2 signal pathway leads to vigorous tumor cell chemotaxis and proliferation via a motility factor (heregulin-a) released by normal epidermal keratinocytes [6, 7]. HER2 receptor itself is a target for antibody therapy with trastuzumab, a humanized monoclonal anti-HER2 antibody. Very recently, a patient suffering from EMPD of the scrotum with regional lymph node metastasis was treated with wide lesion excision combined with a regimen of trastuzumab 600 mg at 3-week intervals. After 4 courses of the regimen, the metastatic lymph nodes of the retroperitoneal and iliac artery partly regressed and 15 months after diagnosis, the patient was alive [8].

Pagetoid epidermal spread is uncommon in apocrine carcinoma of the scrotum and has a unique histological profile. It confirms suspicion of a primary lesion, but a cutaneous metastasis from internal or mammary malignancy cannot be excluded. It requires clinical and radiological exclusion of primary foci. Prolonged and thorough follow-up is mandatory. A definite and precise chemo- or radiotherapy regimen has not been established, beyond wide local excision.
